# Recent Progress in Nanomaterial-Based Electrochemical Biosensors for Cancer Biomarkers: A Review

**DOI:** 10.3390/molecules22071048

**Published:** 2017-06-24

**Authors:** Baozhen Wang, Uichi Akiba, Jun-ichi Anzai

**Affiliations:** 1 Department of Nutrition and Food Hygiene, School of Public Health, Shandong University, 44 Wenhua Xilu, Jinan 250012, China; bzhenw@hotmail.com; 2 Graduate School of Engineering and Science, Akita University, 1-1 Tegatagakuen-machi, Akita 010-8502, Japan; uakiba@gipc.akita-u.ac.jp; 3 Graduate School of Pharmaceutical Sciences, Tohoku University, Aramaki, Aoba-ku, Sendai 980-8578, Japan

**Keywords:** metal nanoparticle, carbon nanotube, graphene, biosensor, cancer biomarker

## Abstract

This article reviews recent progress in the development of nanomaterial-based electrochemical biosensors for cancer biomarkers. Because of their high electrical conductivity, high affinity to biomolecules, and high surface area-to-weight ratios, nanomaterials, including metal nanoparticles, carbon nanotubes, and graphene, have been used for fabricating electrochemical biosensors. Electrodes are often coated with nanomaterials to increase the effective surface area of the electrodes and immobilize a large number of biomolecules such as enzymes and antibodies. Alternatively, nanomaterials are used as signaling labels for increasing the output signals of cancer biomarker sensors, in which nanomaterials are conjugated with secondary antibodies and redox compounds. According to this strategy, a variety of biosensors have been developed for detecting cancer biomarkers. Recent studies show that using nanomaterials is highly advantageous in preparing high-performance biosensors for detecting lower levels of cancer biomarkers. This review focuses mainly on the protocols for using nanomaterials to construct cancer biomarker sensors and the performance characteristics of the sensors. Recent trends in the development of cancer biomarker sensors are discussed according to the nanomaterials used.

## 1. Introduction

Electrochemical biosensors are analytical devices that are fabricated by modifying the surface of electrodes with biomolecules, such as enzymes, antibodies, and DNA [1,2,3]. These sensors are used in biomedical analysis [4], environmental monitoring [5], and process control in food manufacturing industries [6]. Electrochemical biosensors can be used for determining target molecules in sample solutions without pre-treatment of samples owing to the specific binding or interactions between biomaterials and target molecules. A glucose biosensor is a prototype enzyme biosensor that is constructed by immobilizing glucose oxidase (GOx) on the surface of metal or carbon electrodes [7,8]. Glucose biosensors rely on the GOx-catalyzed oxidation reaction of glucose in sample solutions, such as blood, by which gluconolactone and hydrogen peroxide (H_2_O_2_) are produced. The resulting H_2_O_2_ is oxidized on the electrode to generate an electric current as the sensor output signal. Thus, glucose biosensors are currently widely used for determining blood glucose levels in the treatment of diabetic patients. 

**Figure 1:**
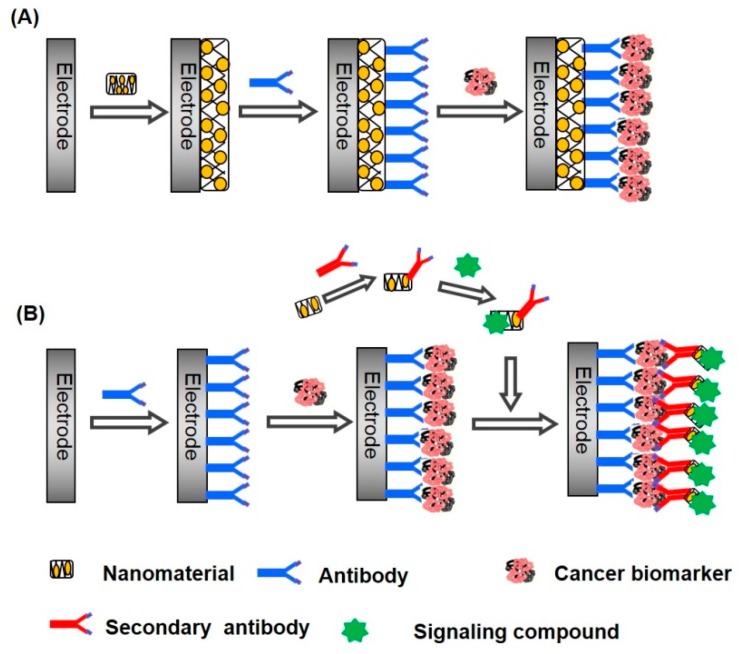
Use of nanomaterials as a surface modifier (**A**) and as a signaling label (**B**) in constructing electrochemical biosensors for cancer biomarkers.

Recently, electrochemical biosensors capable of detecting cancer biomarkers have attracted much attention. Cancer biomarkers are produced and secreted at higher levels from cancerous cells and tissues. Many proteins have been identified as cancer biomarkers, including prostate-specific antigen (PSA), carcinoembryonic antigen (CEA), and α-fetoprotein (AFP) [9,10]. A variety of techniques, such as radio-immunoassay [11], fluorescence spectroscopy [12,13], mass spectroscopy [14,15], and chromatography [16], are available for detecting cancer biomarkers. Although these techniques provide highly sensitive detection of biomarkers in biological fluids, measurements are sometimes tedious and often costly. Therefore, electrochemical biosensors have been intensively studied for developing simple, inexpensive protocols for biomarker detection. A problem in detecting cancer biomarkers is that the concentration of cancer biomarkers is extremely low in biological fluids. The concentrations of cancer biomarkers, such as PSA, CEA, and AFP, are typically several nanograms per milliliter [17,18]. Consequently, highly sensitive biosensors are required for the precise detection of cancer biomarkers. Using metal and carbon nanomaterials as components of biosensors improves the sensitivity of electrochemical biosensors [19,20,21,22,23,24]. Nanomaterials can be used in electrochemical biosensors as surface modifiers of electrodes and as signaling labels. The surface of electrodes is often modified with metal and carbon nanomaterials to enhance effective surface area of the electrodes and to accelerate electron transfer across the electrode surface for amplifying output signals (Figure 1A). In addition, a large amount of proteins can be immobilized on nanomaterial-modified electrodes owing to the high surface area of the electrodes. Alternatively, nanomaterials conjugated with signaling molecules, such as enzymes and redox-active compounds, are often used as labels to enhance the output signals (Figure 1B). In both cases, the high surface area-to-weight ratio of the nanomaterials is exploited. Nanomaterial-based electrochemical biosensors can be operated through different detection modes, such as voltammetry, amperometry, and impedimetry, depending on the types of analytes and nanomaterials used. Enzymes and redox-active compounds are often immobilized on the electrode surface to construct amperometric and voltammetric sensors, while redox ions such as ferricyanide/ferrocyanide ions (Fe(CN)_6_^3−/4−^) are dissolved in the sample solution to record the output signals of impedimetric sensors.

Several papers have reviewed the preparation of biosensors for biomarkers and their use in diagnostic analysis [25,26,27,28]. A variety of biosensors have been constructed using different materials. This paper provides an overview of recent progress made in electrochemical biosensors for cancer biomarkers. Discussion is focused mainly on using metal and carbon nanomaterials for constructing high-performance biosensors for cancer biomarkers based on selected papers published in the past few years.

## 2. Metal Nanoparticle-Based Biosensors for Cancer Biomarkers

Gold nanoparticles (AuNPs) exhibit high conductivity, high affinity and compatibility for biomolecules, and thus AuNPs are the metal nanoparticles most widely used for constructing electrochemical biosensors [29,30]. AuNPs can be prepared by reducing Au(III) to Au(0) with a reducing agent such as sodium borohydride in solution. Two different protocols have been employed in constructing AuNP-based biosensors, as illustrated in Figure 1. AuNPs have been used to modify the surface of sensor electrodes to increase the effective surface area of the electrode, which enables a larger number of biomolecules to be immobilized on the electrode. In another route, AuNPs are used as labels for generating electrochemical signals and increasing the intensity of the signals. The AuNP labels are often conjugated with signaling molecules such as enzymes and redox-active compounds. In this section, AuNP-based electrochemical biosensors for cancer biomarkers are grouped into the above two categories and we discuss the preparing and performance characteristics of the sensors.

### 2.1. AuNP-Modified Electrodes as Biosensors

**Figure 2:**
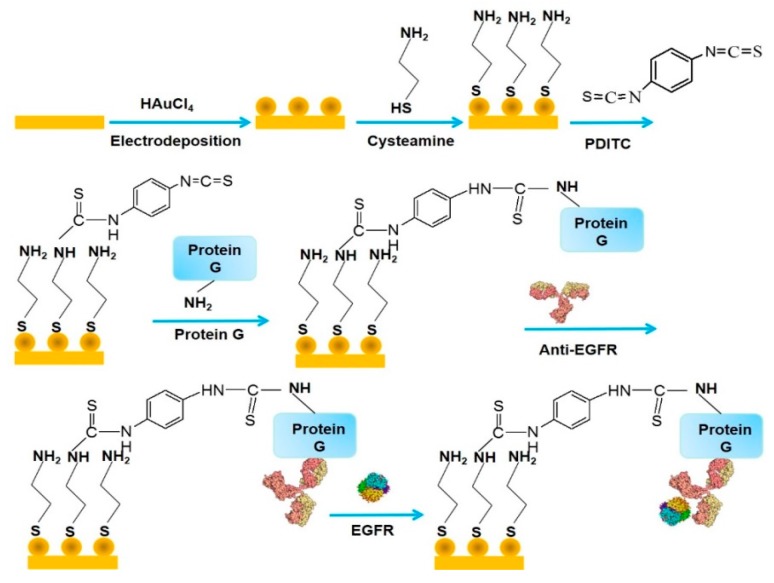
Protocol for preparing epidermal growth factor receptor (EGFR) sensors through stepwise deposition of cysteamine, *p*-phenyldiisothiocyanate (PDITC), protein G, and anti-EGFR on a gold nanoparticle (AuNP)-modified electrode. Reprinted with permission from [33]. Copyright 2013, Elsevier.

The surface of electrodes can be modified with AuNPs through different procedures. Electrodeposition of AuNPs is a simple way to prepare AuNP-modified electrodes. Typically, AuNPs were deposited on the surface of a glassy carbon (GC) electrode by applying a constant potential at −0.4 V vs. Ag/AgCl (3 M KCl) in 5 mM HAuCl_4_ solution in 0.1 M KNO_3_ [31]. The AuNP-modified GC electrode was further modified with protein A and AFP antibody (anti-AFP) to prepare immunosensors for AFP, a biomarker for liver cancer. A sandwich immunoassay using anti-AFP conjugated with horseradish peroxidase (HRP) could detect AFP in serum in the range of 5–80 ng mL^−1^. A similar protocol provided highly sensitive AFP sensors that showed response to AFP in the range of 0.005–0.2 ng mL^−1^ [32]. In another study, impedimetric immunosensors for epidermal growth factor receptor (EGFR) were studied by using an AuNP-modified Au electrode [33]. EGFR is a protein over-expressed in epithelial tumors, including breast, gastric, colorectal, renal, pancreatic, and ovarian cancers. AuNPs were deposited on the surface of an Au electrode by scanning the electrode potential from −0.2 to −1.2 V for 20 cycles in 6 mM HAuCl_4_ solution in 0.1 M KNO_3_. The EGFR sensors were prepared by modifying the AuNP-modified electrode with protein G and anti-EGFR antibody (Figure 2). The dynamic range of the impedimetric response of the sensor to EGFR was from 1 pg mL^−1^ to 1 μg mL^−1^. 

**Figure 3:**
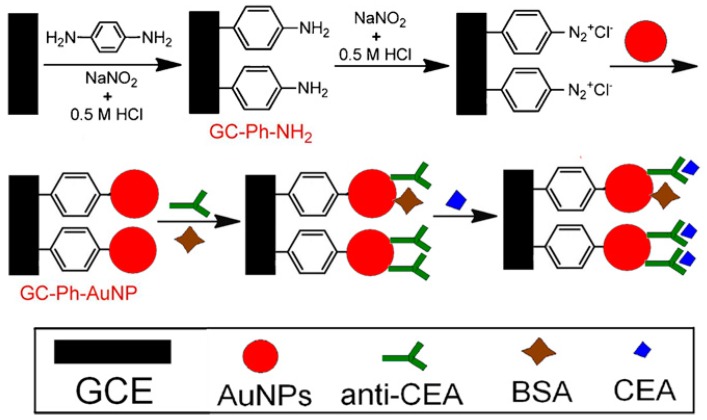
Deposition of as-prepared AuNPs on a glassy carbon (GC) electrode (GCE) for preparing the carcinoembryonic antigen (CEA) immunosensor. Reprinted with permission from [36]. Copyright 2012, Elsevier.

Another route for constructing AuNP-modified electrodes is to deposit as-prepared AuNPs on electrodes by methods such as dip-coating, drop-casting, or multilayer deposition. In this procedure, the surface of the electrodes is first coated with self-assembled molecular monolayers or polymer films to facilitate the AuNP deposition. For example, the surface of a GC electrode was coated with an electro-polymerized poly(2,6-pyridinediamine) film, followed by the deposition of AuNPs by dip-coating [34]. Immunosensors sensitive to a prostate cancer marker, PSA, were prepared by immobilizing anti-PSA on the AuNPs. To read out the signal, a ferrocene-tagged label was used during voltammetry. The immunosensor exhibited a response to PSA over a concentration range from 2.0 pg mL^−1^ to 10 ng mL^−1^, with a lower detection limit of 0.5 pg mL^−1^. Amino group-bearing cross-linked polyethylene glycol was also used as a surface coating for depositing AuNPs on GC electrodes [35]. The AuNP-deposited GC electrode was further modified with single-strand 19-mer oligonucleotides to construct DNA biosensors for detecting the breast cancer susceptibility gene BRCA1. The hybridization of the BRCA1 sequence on the electrode surface induced changes in the electrochemical impedance spectroscopy. The BRCA1 sensor exhibited a linear response to BRCA1 from 50 fM to 1 nM, with a lower detection limit of 1.72 fM. A phenyldiazonium salt-modified electrode was also used for depositing AuNPs by taking advantage of the high binding energy of C-Au bonds [36] (Figure 3). The AuNP-immobilized electrode was modified with anti-CEA to construct CEA immunosensors. The CEA sensors showed a linear response to CEA in a concentration range from 10 fg mL^−1^ to 100 ng mL^−1^, which was evaluated by recording the voltammetric current of Fe(CN)_6_^3−/4−^ in solution. Fe(CN)_6_^3−/4−^ has been widely used as redox indicator in voltammetric sensors because the accessibility of Fe(CN)_6_^3−/4−^ to the electrode surface is greatly suppressed or enhanced depending on the chemical events occurring on the electrode surface [37,38,39].

It is not always necessary to modify the electrode surface with a monolayer or polymer films before depositing AuNPs. For example, AuNPs were deposited directly on the surface of an indium tin oxide (ITO) electrode by dip-coating [40]. The deposited AuNPs were modified with anti-heat shock protein 70 (anti-HPS70). HPS70 overexpression is a potential marker of prostate, breast, and pancreatic cancers. The immunosensor exhibited an impedimetric response to HPS70 in a concentration range of 1–166 fg mL^−1^. Inkjet-printed AuNP electrodes were also useful as base electrodes for biomarker sensors [41]. In this protocol, AuNP ink was prepared by dissolving dodecanethiol-protected AuNPs in toluene (100 mg mL^−1^) and was printed onto a flexible polyimide film.

The layer-by-layer (LbL) deposition technique has been attracting much attention for constructing biosensors [7,42,43,44]. LbL deposition relies on the alternate deposition of two kinds of polymers or biomolecules from solutions onto solid substrates through electrostatic, hydrogen-bonding, and biological interactions. According to this protocol, AuNP thin films were deposited on the surface of microfluidic electrochemical sensors by using glutathione-coated AuNPs and poly(diallyldimethylammonium chloride) [45,46]. Microfluidic immunosensors for several cancer biomarkers, such as PSA and interleukin-6 (IL-6), were prepared by immobilizing the corresponding antibodies on the AuNPs. The microfluidic sensor array was useful for simultaneous detection of the cancer biomarkers at sub-picogram per milliliter levels in serum.

It is possible to use AuNP-containing nanocomposites as a surface modifier of electrodes for constructing electrochemical biosensors. AuNP-graphene-silica sol-gel composites were prepared by mixing HAuCl_4_, graphene oxide, and tetraethyl orthosilicate in ethanol and drop-casting the mixture on an ITO electrode [47]. The modified ITO electrode was used to construct IL-6 sensors. In another example, nanocomposites consisting of AuNPs, poly(dopamine), and thionine (Th) were drop-cast on the surface of a GC electrode [48]. Silver nanoparticle (AgNP)-chitosan composites were also used to construct voltammetric sensors for detecting the epithelial cancer biomarker, EpCAM [49]. The immunosensors were used to detect 2.7 pg mL^−1^ EpCAM by using HRP-conjugated secondary antibody as label. Highly stable surface architectures were prepared on a Au electrode by successively covalently attaching amino-functionalized silica-coated AuNPs, carboxyl-terminated cadmium selenide (CdSe) quantum dots (QDs), and an antibody for the ovarian cancer biomarker carbohydrate antigen-125 (CA-125) [50]. The biosensor showed an electrochemical impedimetric response to CA-125 in the range of 0–0.1 U mL^−1^ with a detection limit of 0.0016 U mL^−1^. The CA-125 sensor had a high reproducibility, probably due to the covalent linkage of the nanomaterials on the electrode surface.

### 2.2. Metal Nanoparticles as Signaling Labels

A problem in constructing electrochemical immunosensors is that no electric signal can be obtained from the immune reaction itself. This is because immuno-complexation does not produce redox-active products, unlike enzymatic reactions. Therefore, electrochemical immunosensors must be coupled with redox-active compounds to obtain output signals. Nanocomposites that consist of metal nanoparticles, secondary antibodies, and signaling compounds, such as enzymes, QDs, and redox dyes, have been used. 

**Figure 4:**
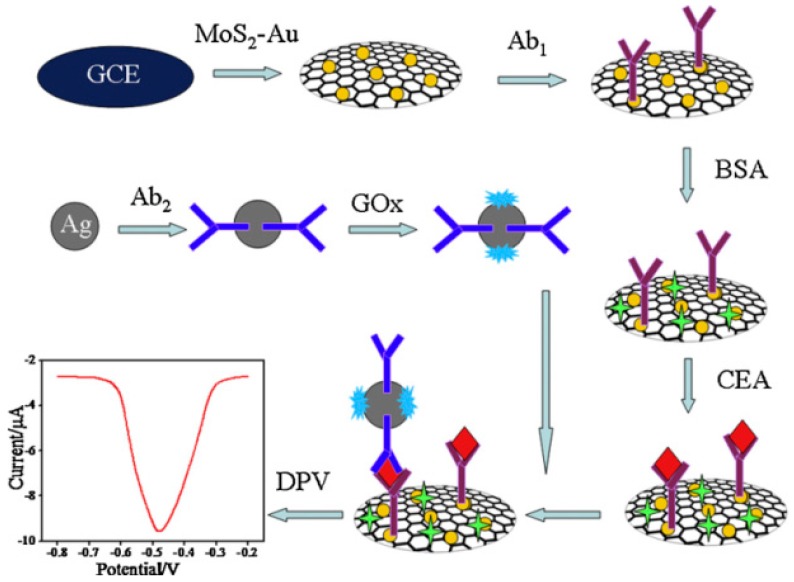
Preparation of the CEA immunosensor with Ag nanoparticles modified with a secondary antibody and glucose oxidase (GOx) as a signaling label. Reprinted with permission from [54]. Copyright 2015, Elsevier.

**Figure 5:**
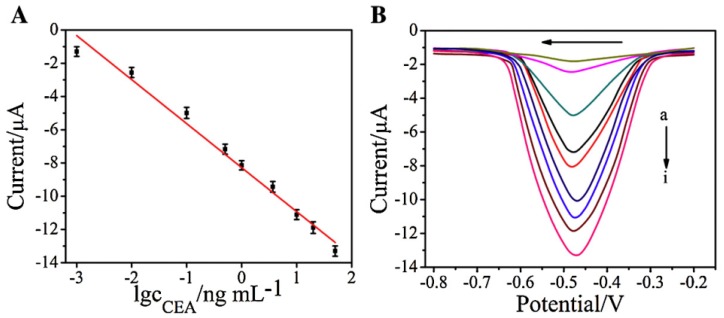
Calibration graph for the CEA sensor (**A**) and differential pulse voltammetry (DPV) (**B**). Reprinted with permission from [54]. Copyright 2015, Elsevier.

Enzymes are often used as signaling labels because enzymatic reactions can be coupled with reactions that consume or produce redox-active compounds. For instance, nanocomposites composed of AuNPs, AFP, and HRP were assembled on the surface of porous zinc oxide (ZnO) particles and used as a signaling label in electrochemical AFP sensors [51]. The competitive binding of AFP and the signaling label to an anti-AFP-modified electrode provided a surface loaded with the signaling label, the density of which depended on the concentration of AFP. After competitive binding, the electrode was incubated in a solution of 4-chloro-1-napththol (4CN) solution and H_2_O_2_ to deposit insoluble benzo-4-chlorohexadienone (B4CH) produced by HRP. Thus, the redox reaction of Fe(CN)_6_^3−/4−^ on the electrode was blocked by B4CH depending on the concentration of AFP in the sample. This sensor showed a response to AFP in a concentration range of 0.2 pg mL^−1^ to 500 ng mL^−1^, with a detection limit of 0.08 pg mL^−1^. Nanocomposites assembled with AuNPs and HRP were also used in other studies as a signaling enzyme [52,53]. In another study, nanocomposites containing GOx were used in immunosensors for CEA [54] (Figure 4). The output signal of this sensor was detected by differential pulse voltammetry (DPV) recorded in glucose solution (Figure 5). 

The redox activity of AgNPs makes them useful as signaling labels for electrochemical sensors. AgNPs that were conjugated with ZnO nanospheres and a secondary antibody for PSA were used as signaling labels in PSA sensors [55]. The output signal of the PSA sensor was obtained through the AgNP-catalyzed reduction current of H_2_O_2_ in cyclic voltammetry (CV). The redox reactions of AgNPs could be used to record the output signal of the sensors because Ag can be electrochemically oxidized to Ag_2_O. Ag-Au composite nanoparticles have been used as redox-active labels in CEA sensors [56]. Furthermore, the electrochemical stripping current of nanoparticles, such as Au and Au-Ag composites, can be used to record the signals of sandwich immunosensors. Based on this protocol, PSA and EGFR were detected [57,58]. These examples demonstrated that nanoparticles with redox activity are useful as signaling labels in electrochemical immunosensors. In this context, redox-active organometallic compounds can be used for constructing signaling labels. Iron oxide (Fe_3_O_4_) nanocomposites modified with anti-PSA and ferrocene were used as redox label in sandwich immunosensors for PSA [59]. The PSA sensors exhibited a redox current originating from the ferrocene moieties in the sandwich immunoassay. Thus, because of their high stability, versatile structures, and low cost, ferrocene derivatives are useful in preparing redox-active nanocomposites as signaling labels in biosensors [60,61].

QDs have been used as labels for increasing the output signals of biosensors. QDs are nanoparticles consisting of 10–50 atoms of semiconducting materials with diameters of several nanometers [62]. QDs show unique optical and electronic properties owing to their small size and high surface area-to-weight ratios. Electrochemical immunosensors were constructed using QDs for assaying Golgi protein 73 (GP73), a biliary tract cancer biomarker [63]. This sensor was coupled with CdSe QD-tagged lectin as a label, in which the lectin specifically bound to the hydrocarbon chains of GP73. The sensor exhibited a detection limit of 12 pM in direct serum analysis. As shown in this example, biomarker proteins often contain hydrocarbon chains on the molecular surface, enabling a sandwich immunoassay using lectins [64,65]. Thus, using boronic acid-modified synthetic lectins (or boronolectins) as a recognition element for biomarker proteins may be promising in constructing biosensors because boronic acids bind specifically to hydrocarbon chains of biomarker proteins [66,67,68]. CdSe QDs were also used in photoelectrochemical sensors for CEA coupled with nanocomposites composed of a secondary antibody and CuO nanoparticles [69]. Table 1 summarizes the performance characteristics of the metal nanoparticle-based cancer biomarker sensors discussed above.

## 3. Carbon Nanotube-Based Biosensors for Cancer Biomarkers

Carbon nanotubes (CNTs) are carbon molecules with a cylindrical hollow structure having walls formed by one-atom-thick sheets of sp^2^-hybridized carbon. The diameter of CNTs is typically 0.5–50 nm and the length is usually several micrometers. In an extreme case, CNTs 18.5 cm long have been reported [70]. CNTs are categorized as single-wall carbon nanotubes (SWCNTs) and multi-walled carbon nanotubes (MWCNTs) depending on the number of layers of sheets in the wall. Owing to their high mechanical strength and electrical and thermal conductivity, CNTs are widely used in constructing nano-devices including biosensors [71,72,73,74].

### 3.1. CNT-Modified Electrodes as Biosensors

The advantages of CNT-modified electrodes include an increased surface area with high conductivity and the possibility of chemically modifying the surface. Consequently, CNT-modified electrodes have been widely used for constructing high-performance biosensors for cancer biomarkers.

**Figure 6:**
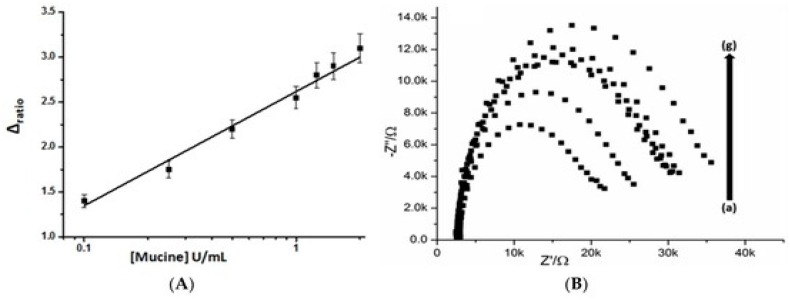
(**A**) A calibration graph of aptamer sensor for the mucine. The changes in electron transfer resistance (∆_ratio_) were plotted vs. mucine concentration; (**B**) Nyquist plots of the aptamer sensor in the presence of mucine from 0.1 (a) to 2.0 U mL^−1^ (g). Reprinted from [76]. Copyright 2016 MDPI.

Screen-printed SWCNT electrodes were used for constructing label-free immunosensors for human chorionic gonadotropin (hCG) by modifying the surface of the electrode with an anti-hCG antibody [75]. hCG is a diagnostic marker for pregnancy as well as for ovarian and testicular cancers. The response of the sensors depended on the concentration of hCG in the concentration range from 0.01 to 100 ng mL^−1^. In another study, conventional screen-printed carbon electrodes were modified with MWCNTs to prepare aptamer sensors for the breast cancer marker mucine (MUC1) [76]. The aptamer sensors showed an impedimetric response to MUC1 in a range of 0.1–2 U mL^−1^ (Figure 6). In addition, CEA biosensors were fabricated from conductive paper (6.5 × 10^−4^–2.2 × 10^−4^ S cm^−1^), which was prepared by doping a filter paper 0.18-mm-thick with carboxylated CNTs by dip-coating [77]. The surface of the conductive paper was then covalently modified with the CEA antibody (anti-CEA) through carbodiimide coupling to construct CEA sensors. This paper-based sensor exhibited an impedimetric response to CEA in the physiological range (2–15 ng mL^−1^).

Voltammetric detection of cancer biomarkers has been studied based on CNT-modified electrodes. A GC electrode was coated with nanocomposites of carboxylated MWCNTs followed by covalent modification by a lectin specific to α2,3-sialylated glycans [78]. The concentration of α2,3-sialylated glycans in serum is a diagnostic marker for carcinoma apoptosis as well as tumors. The binding of α2,3-sialylated glycans to the electrode surface was detected through the DPV response. This sensor exhibited high sensitivity to α2,3-sialylated glycans in the concentration range of 10 fg mL^−1^ to 50 ng mL^−1^, with a detection limit of 3 fg·mL^−1^. To improve sensitivity of biomarker sensors, MWCNT-embedded highly oriented ZnO nanowires were synthesized by electrospinning [79]. The ZnO nanowires were coated on a silicon substrate and the surface was modified with an antibody for CA-125 as an ovarian cancer biomarker. The electrochemical activity of MWCNT-embedded ZnO nanowires was much higher than that of pure ZnO nanowires. Another approach includes using vertically aligned SWCNT arrays (or CNT forests) to modify the electrode surface [80]. The SWCNTs was modified with an antibody for matrix metalloproteinase-3 (MMP-3) as a biomarker for squamous cell carcinoma and adrenal tumors. Based on a sandwich immunoassay with HRP labels, this sensor exhibited a detection limit of 4 pg mL^−1^ for MMP-3. Chemiluminescence biosensors for PSA and IL-6 were also prepared based on CNT forests [81].

**Figure 7:**
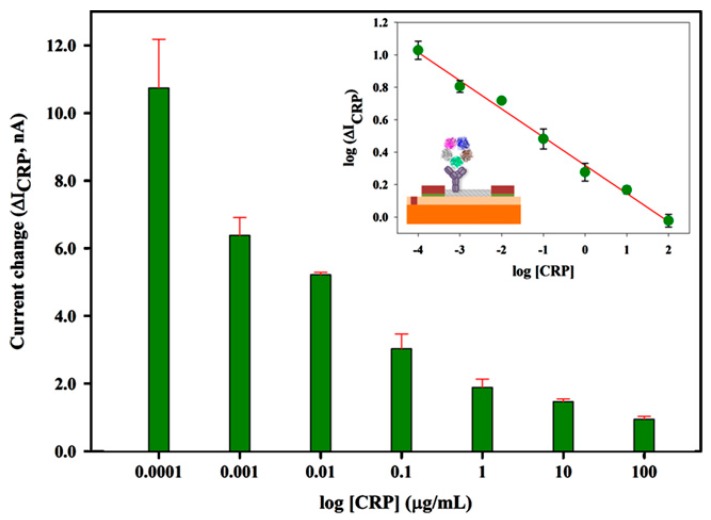
Changes in drain current of an field effect transistors (FET) immunosensor for C-reactive protein (CRP). Inset shows a schematic illustration of the CRP sensor and its calibration graph. Reprinted with permission from [85]. Copyright 2013, Elsevier.

Preliminary studies demonstrated that CNT-modified field effect transistors (FETs) are promising devices as platforms for constructing biosensors [82,83]. Recently, a CNT network was grown via chemical vapor deposition on the surface of a silicon wafer to fabricate CNT-modified FETs for detecting the prostate cancer marker osteopontin (OPN) [84]. The CNT-modified FETs showed current-gate voltage characteristics that depended on the concentration of OPN from 0.001 to 1000 ng mL^−1^. An advantage of FET sensors is that the electric response can be recorded under dry conditions without sample solutions. For instance, Justino and coworkers prepared C-reactive protein (CRP) sensors using CNT-modified FETs, for which the drain current was measured under dry conditions after exposing the sensors to CRP solutions [85] (Figure 7). This is advantageous in eliminating the potential effects of ionic species in sample solutions.

### 3.2. CNTs as Signaling Labels

**Figure 8:**
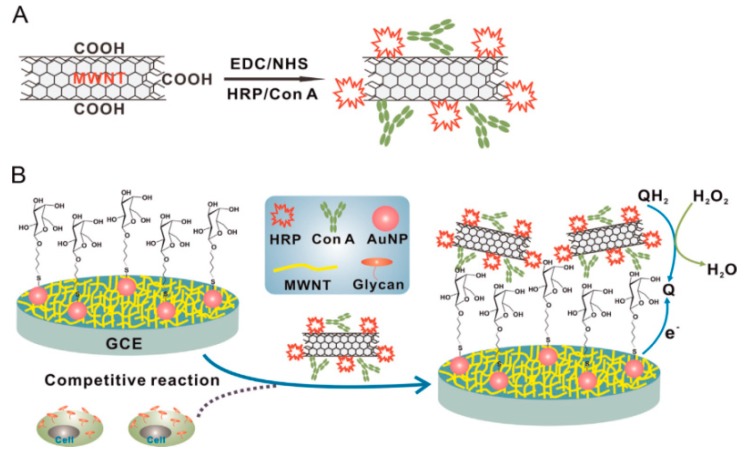
Preparation of multi-walled carbon nanotube (MWCNT) labels modified with horseradish peroxidase (HRP) and Con A (**A**) and protocol for determining glycan expression on cancer cells (**B**). Reprinted with permission from [86]. Copyright 2013, Elsevier.

**Figure 9:**
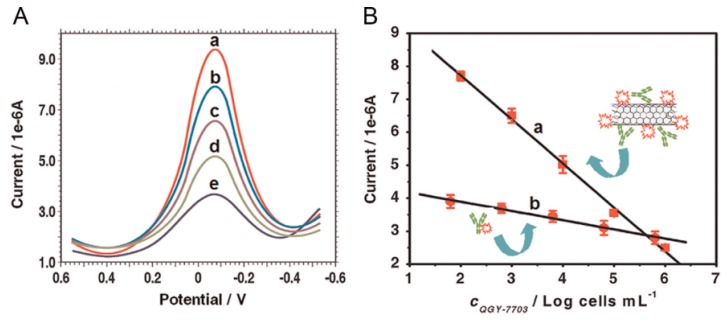
DPV of QGY-7703 (**A**) and its calibration graphs obtained by using the MWCNT and MWCNT-free labels (**B**). Reprinted with permission from [86]. Copyright 2013, Elsevier.

CNTs can be used as a scaffold for preparing signaling labels because of their high surface area-to-volume ratio. Glycan expression on cancer cells was detected by using MWCNT labels modified with HRP and lectin concanavalin A (Con A) [86] (Figure 8). The MWCNT label was prepared by covalently modifying the surface of carboxylated MWCNTs after carbodiimide activation. In the competitive binding of target cancer cells and the MWCNT label, the mannose-modified GC electrode was immersed in the sample solutions containing cancer cells and the labels. The GC electrode was then immersed in a solution of H_2_O_2_ and hydroquinone as substrates of HRP to record the output signals in DPV. The output signals of the sensor were inversely proportional to the concentration of cancer cells in samples. Thus, human liver cancer cells (QGY-7703) were detected with a detection limit of 40 cells mL^−1^ (Figure 9). MWCNT labels modified with ferritin and a secondary antibody were also used as labels for increasing the sensitivity of immunosensors for carbohydrate antigen 15-3 (CA153) as a breast cancer marker [87].

In a similar protocol, PSA sensors based on Au electrodes were coupled with MWCNT labels [88]. The MWCNT labels were prepared by covalent bonding of anti-PSA and HRP to carboxylated MWCNTs. After sandwich binding of the MWCNT labels to the PSA captured on the electrode, the electrode was incubated in a solution of 4CN and H_2_O_2_ to deposit insoluble B4CH through HRP catalysis on the electrode surface. The redox reaction of Fe(CN)_6_^3−/4−^ on the electrode was blocked by B4CH depending on the concentration of PSA in the sample. The linear response range of the PSA sensor was 1 pg mL^−1^ to 10 ng mL^−1^ and the detection limit was 0.4 pg mL^−1^. The high sensitivity of the sensor was explained by the multiple HRP binding to MWCNTs, compared with the results obtained by using labels without MWCNTs. A similar study of AFP sensors based on labels made of carbon nanohorns (CNHs) has been reported [89]. CNHs are carbon nanomaterials with a tubular structure similar to SWCNTs, except that CNHs are characterized by a long cone-shaped tip. CNH labels were prepared by modifying carboxylated CNHs with anti-AFP, HRP, and GOx through carbodiimide coupling. The electrochemical signal of this sensor was obtained either from CV or impedimetric spectroscopy in glucose solutions. Under optimal conditions, the AFP sensors showed a linear response range from 0.001 to 60 ng mL^−1^, with a detection limit of 0.33 pg mL^−1^. CNH labels modified with Au and a secondary antibody for AFP were also used in AFP immunosensors [90]. A screen-printed carbon electrode was first modified with anti-AFP, and then incubated in the sample solution containing AFP and a known concentration of the CNH label to bind target AFP and the label competitively. The electrochemical signals of this sensor were obtained from the oxidation current of Au in the label in 0.1 M HCl solution. The detection limit of the sensor was 0.07 pg mL^−1^. Table 2 summarizes the performance characteristics of carbon nanotube-based cancer biomarker sensors discussed above.

## 4. Graphene-Based Biosensors for Cancer Biomarkers

Graphene is a two-dimensional one-atom-thick sheet consisting of sp^2^-hydridized carbon atoms. Thus, graphene is a structural component of CNTs, in which graphene sheets are rolled into cylinders with nanometer-sized diameters. Graphene exhibits excellent mechanical strength and high electrical conductivity, similar to CNTs. Therefore, graphene has been used extensively as electrode modifiers and signaling labels in electrochemical biosensors [91,92,93,94].

### 4.1. Graphene-Modified Electrodes

Recently, two groups have independently constructed hCG sensors based on screen-printed graphene electrodes and compared their performance characteristics with hCG sensors prepared using conventional electrodes. Ahmed and coworkers used commercially available screen-printed graphene electrodes to prepare hCG sensors by immobilizing anti-hCG through physical adsorption [95]. This sensor showed a linear response to hCG in the concentration range of 5–500 pg mL^−1^, with a detection limit of 5 pg mL^−1^. The detection limit of this hCG sensor was substantially lower than those of hCG sensors based on carbon- and CNT-based screen-printed electrodes (36 and 13 pg mL^−1^, respectively [96,97]). On the other hand, Sales and coworkers reported a label-free hCG sensor based on a screen-printed graphene electrode coated with poly(aniline) film [98]. This sensor could detect hCG from 0.001 to 50 ng mL^−1^ in a urine sample with a detection limit of 0.286 pg mL^−1^ by impedimetric assay. Both studies demonstrated that screen-printed graphene electrodes are useful for constructing highly sensitive hCG sensors. Sales and coworker claimed that using graphene without covalent modification resulted in the excellent electrical properties of the sensor. In addition, the high sensitivity of the hCG sensor was ascribed to the oriented immobilization of antibodies on the surface of the electrode through the covalent linkage of antibody proteins to the poly(aniline) film.

Paper-based microfluidic immunosensors for cancer biomarkers were developed using reduced graphene oxide (rGO) [99,100,101]. Microfluidic electrochemical devices were fabricated on a cellulose paper by photolithography, in which eight working electrodes and reference and counter electrodes were screen printed with carbon ink [99]. The working electrodes were modified with a drop-cast GO dispersion followed by electrochemical reduction to rGO to allow further modification with antibodies. According to this protocol, four kinds of antibodies for AFP, CEA, CA125, and CA153 were immobilized on the working electrodes. This sensor exhibited voltammetric responses to AFP, CEA, CA125, and CA153 in the concentration ranges of 0.001–100, 0.005–100, 0.001–100, and 0.005–100 ng mL^−1^, respectively. Paper-based devices are promising as a platform for developing low-cost, environmentally friendly biosensors.

Electrodes were modified with several graphene derivatives including *N*-doped rGO [102], epitaxially grown multilayered GO [103], and three-dimensional macro-porous GO foams [104] to improve the performance of cancer biomarker sensors. 

**Figure 10:**
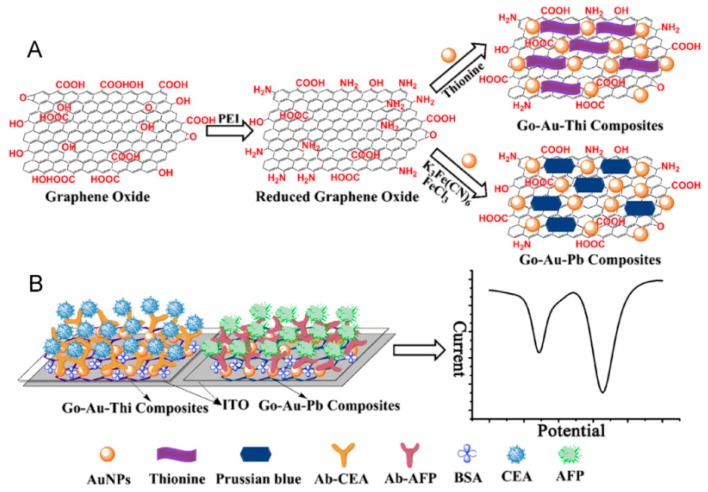
Preparation of reduced graphene oxide (rGO) modified with thionine (TH) and Prussian blue (PB) (**A**) and CEA and AFP sensors based on rGO-modified electrodes (**B**). Reprinted with permission from [105]. Copyright 2014, Elsevier.

Graphene is often combined with other redox-active compounds to improve their electrochemical activity. For example, TH and Prussian blue (PB) were coupled with rGO in constructing CEA and AFP sensors, respectively [105] (Figure 10). rGO/TH and rGO/PB composites were further conjugated with AuNPs to form rGO/AuNPs/TH and rGO/AuNPs/PB for surface modification of ITO electrodes. The detection limits of these CEA and AFP sensors were more than one order of magnitude lower than those of other CEA and AFP sensors [106]. In another study, GC electrodes modified with a thin film of GO/cobalt hexacyanoferrate nanocomposites were used for constructing PSA sensors [107]. The redox signals of this sensor was approximately 10 times higher than those of sensors prepared without GO, which clearly showed the role of GO in improving the redox signals of the PSA sensor. Nanocomposites composed of rGO and zirconia (ZrO_2_) were also used as surface modifiers for ITO electrodes to prepare biosensors for oral cancer biomarker CYFRA-21-1 [108]. The rGO/ZrO_2_-modified ITO electrode showed electron transfer kinetics 2 times higher than the rGO-free ZrO_2_/ITO electrode. In addition, in other studies, ionic liquid-functionalized GO and GO-containing cryogels have been used to construct cancer biomarker sensors [109,110].

### 4.2. Graphene As Signaling Labels

Owing to its high surface area and versatility for surface modifications, graphene has been used as a scaffold for constructing signaling labels for electrochemical immunosensors. Ma and colleagues prepared signaling labels for simultaneous detection of CEA and AFP on a single probe [111]. In these labels, carboxylated GO sheets were modified with toluidine blue (TB) and anti-CEA or PB and anti-AFP, respectively. The redox signals of modified GO sheets in DPV, which originated from redox reactions of TB and PB, were recorded in a sandwich immunoassay. These sensors could detect CEA and AFP down to 0.1 and 0.05 ng mL^−1^, respectively. Cross-reactivity between CEA and AFP was negligible in the simultaneous detection of the two analytes. The same group also used GO/PB/Au/ionic liquid nanocomposites as signaling labels for the ultrasensitive detection of AFP [112]. Thus, the detection limit of the sensor was improved to 4.6 pg mL^−1^. Redox catalysts such as copper sulfide (CuS) were deposited on the GO sheets through in situ growth to prepare signaling labels for AFP sensors [113]. The catalytic current originating from the CuS/Cu_2_S redox couple on the GO sheets increased in the presence of H_2_O_2_ depending on the concentration of AFP in a range of 0.001 to 10 ng mL^−1^. GO nanocomposites have been used as signaling labels in biomarker sensors and in other biosensors including H_2_O_2_ sensors, bacteria sensors, and drug sensors [114,115,116,117,118,119]. Table 3 summarizes the performance characteristics of graphene-based biosensors for cancer biomarkers discussed above.

## 5. Conclusions

Metal nanoparticles and carbon nanomaterials have been used for preparing electrochemical biosensors for cancer biomarkers. Nanomaterials are used to modify the surface of electrodes to enhance effective surface area of the electrodes and to accelerate electron transfer across electrode/solution interfaces, which increases the output signals of biosensors. Furthermore, the electrochemical signals of biosensors can be increased by using signaling labels consisting of nanomaterials and secondary antibodies. In both cases, the high surface area-to-weight ratio and facile surface modification of the nanomaterials are exploited. Thus, a variety of biosensors has been developed by using nanomaterials coupled with antibodies specific to cancer biomarkers. Nanomaterial-based biosensors are useful for detecting cancer biomarkers in extremely low concentration ranges, including picograms per milliliter. A drawback of most biosensors cited in this article relates to the fact that they require multi-step measurements consisting of immune complexation of targets followed by electrochemical measurements. Therefore, a challenge in developing cancer biomarker sensors is developing protocols for single-step measurements. Another problem may arise from somewhat complicated and multi-step procedures in the modification of electrode surface for the construction of biosensors. A simpler protocol for assembling nanomaterials as well as biomolecules on the surface of electrode is necessary.

Most of the biosensors discussed in this article allow highly sensitive and selective detection of cancer biomarkers owing to the use of nanomaterials as components. Therefore, the biosensors would be highly useful as key elements for the development of automated diagnostic systems. For this goal, however, the performance characteristics of biosensors, including reusability, stability, and compatibility with biological fluids, should be further improved. Electrochemical biosensors could be used in clinical laboratories and hospitals for the low-cost diagnosis and management of cancers if these problems are solved.

## Figures and Tables

**Table 1:** Metal nanoparticle-based biosensors for cancer biomarkers.

Nanomaterials Used	Electrode	Transduction Method	Analyte	Detection Range	LOD	Ref.
protein A/AuNPs	GCE	voltammetry	AFP	5–80 ng mL^−1^	3.7 ng mL^−1^	[31]
protein G/AuNPs	AuE	voltammetry	AFP	0.005–0.2 ng mL^−1^	2 pg mL^−1^	[32]
protein G/AuNPs	AuE	impedimetry	EGFR	0.001–1000 ng mL^−1^	0.34 pg mL^−1^	[33]
ferrocene/AuNPs	GCE	voltammetry	PSA	0.002–10 ng mL^−1^	0.5 pg mL^−1^	[34]
DNA/AuNPs	GCE	impedimetry	BRCA1	50 fM–1 nM	1.72 fM	[35]
AuNPs	GCE	voltammetry	CEA	10 fg mL^−1^–100 ng mL^−1^	3 fg mL^−1^	[36]
AuNPs	ITO	impedimetry	HSP70	1–166 fg mL^−1^	0.0618 fg mL^−1^	[40]
glutathione/AuNPs	SPCE	amperometry	IL-6	0.3–20 pg mL^−1^	0.3 pg mL^−1^	[45]
glutathione/AuNPs	SPCE	amperometry	PSA	0.225–5 pg·mL^−1^	0.1 pg mL^−1^	[45]
graphene/AuNPs	ITO	amperometry	IL-6	1–40 pg mL^−1^	0.3 pg mL^−1^	[47]
Th/PDA/GO/AuNPs	GCE	voltammetry	AFP	0.1–150 ng mL^−1^	0.03 ng mL^−1^	[48]
CdSe/silica/AuNPs	AuE	impedimetry	CA-125	0–0.1 U mL^−1^	0.0016 U mL^−1^	[50]
ZnO/AuNPs	GO paper	voltammetry	AFP	0.0002–500 ng mL^−1^	0.08 pg mL^−1^	[51]
GOx/AgNPs	GCE	voltammetry	CEA	0.001–50 ng mL^−1^	0.27 pg mL^−1^	[54]
ZnO/AgNPs	AuNRs paper	voltammetry	PSA	0.004–60 ng mL^−1^	1.5 pg mL^−1^	[55]
Ag/AuNPs	GCE	voltammetry	CEA	0.01–120 ng mL^−1^	8 pg mL^−1^	[56]
MCF/AuNPs	GCE	voltammetry	CEA	0.05–1000 pg mL^−1^	0.024 pg mL^−1^	[57]
antibody/AuNPs	GCE	voltammetry	EGFR	1–40 ng mL^−1^	50 pg mL^−1^	[58]
ferrocene/Fe_3_O_4_	GCE	voltammetry	PSA	0.01–40 ng mL^−1^	2 pg mL^−1^	[59]
CdSe QDs	GCE	voltammetry	GP73	20–5000 pM	12 pM	[63]

LOD: lower limit of detection, AuNPs: gold nanoparticles, Th: thionine, PDA: poly(dopamine), GO: graphene oxide, GOx: glucose oxidase, MCF: mesoporous carob foam, QDs: quantum dots, GCE: glassy carbon electrode, AuE: gold electrode, ITO: indium tin oxide electrode, AuNR: gold nanorods, AFP: α-fetoprotein, EGFR: epidermal growth factor receptor, PSA: prostate-specific antigen, BRCA1: breast cancer susceptibility gene, CEA: carcinoembryonic antigen, HSP70: heat shock protein 70, IL-6: interleukin-6, CA-125: carbohydrate antigen-125, GP73: Golgi protein 73.

**Table 2:** Carbon nanotube-based biosensors for cancer biomarkers.

Nanomaterials Used	Electrode	Transduction Method	Analyte	Detection Range	LOD	Ref.
SWCNTs	SPE	impedimetry	hCG	0.01–100 ng mL^−1^	-	[75]
aptamer/MWCNTs	SPE	impedimetry	mucine	0.1–2 U mL^−1^	0.02 U mL^−1^	[76]
PEDOT/CNTs	filter paper	amperometry	CEA	2–15 ng mL^−1^	1 ng mL^−1^	[77]
MWCNTs	GCE	voltammetry	si-Gly	10 fg·mL^−1^–50 ng mL^−1^	3 fg mL^−1^	[78]
MWCNTs/ZnO	GCE	voltammetry	CA125	0.001–1000 U mL^−1^	0.00113 U mL^−1^	[79]
aligned SWCNTs	PGE	amperometry	MMP-3	4–300 pg mL^−1^	4 pg mL^−1^	[80]
antibody/CNTs	FET	*I-V_G_*	OPN	0.001–1000 ng mL^−1^	1 pg mL^−1^	[84]
SWCNTs	FET	*I_D_-V_D_*	CRP	0.0001–100 μg mL^−1^	0.1 ng mL^−1^	[85]
Con A/MWCNTs	GCE	voltammetry	QGY-7703	100–100,000 cells mL^−1^	40 cells mL^−1^	[86]
ferritin/MWCNTs	AuE	voltammetry	CA153	0.05–100 U mL^−1^	0.009 U mL^−1^	[87]
HRP/MWCNTs	AuE	voltammetry	PSA	0.001–10 ng mL^−1^	0.4 pg mL^−1^	[88]
GOx/CNHs	GCE	voltammetry	AFP	0.001–60 ng mL^−1^	0.33 pg mL^−1^	[89]
nano Au/CNHs	SPE	voltammetry	AFP	0.1–1000 pg mL^−1^	0.07 pg mL^−1^	[90]

SWCNTs: single-wall carbon nanotube, PEDOT: poly(3,4-ethylenedioxythiophene), SPE: screen-printed electrode, HRP: horseradish peroxidase, CNHs: carbon nanohorn, PGE: pyrolytic graphite electrode, FET: field effect transistor, *V_G_*: gate voltage, *I_D_*: drain current, *V_D_*: drain voltage, hCG: human chorionic gonadotropin, si-Gly: α2,3-sialylated glycans, MMP-3: matrix metalloproteinase-3, OPN: osteopontin, CRP: C-reactive protein, QGY-7730: human liver cancer cell, CA153: carbohydrate antigen 15-3.

**Table 3:** Graphene-based biosensors for cancer biomarkers.

Nanomaterials Used	Electrode	Transduction Method	Analyte	Detection Range	LOD	Ref.
GO	SPGE	voltammetry	hCG	5–500 pg mL^−1^	5 pg mL^−1^	[95]
GO	SPGE	impedimetry	hCG	0.001–50 ng mL^−1^	0.286 pg mL^−1^	[98]
rGO	SPCE	voltammetry	AFP	0.001–100 ng mL^−1^	1 pg mL^−1^	[99]
rGO	SPCE	voltammetry	CEA	0.005–100 ng mL^−1^	5 pg mL^−1^	[99]
rGO	SPCE	voltammetry	CA125	0.001–100 ng mL^−1^	1 pg mL^−1^	[99]
rGO	SPCE	voltammetry	CA153	0.005–100 ng mL^−1^	5 pg mL^−1^	[99]
*N*-doped rGO	GCE	voltammetry	CA153	0.1–20 U mL^−1^	0.012 U mL^−1^	[102]
multilayer GO	GO/SiC	impedimetry	hCG	0.62–5.62 ng mL^−1^	0.62 ng mL^−1^	[103]
graphene	graphene foam	voltammetry	CEA	0.1–750 ng mL^−1^	90 pg mL^−1^	[104]
thionine/GO	ITO	voltammetry	CEA	0.01–300 ng mL^−1^	0.65 pg mL^−1^	[105]
PB/GO	ITO	voltammetry	AFP	0.01–300 ng mL^−1^	0.885 pg mL^−1^	[105]
CoHCF/GO	GCE	voltammetry	PSA	0.02–2 ng mL^−1^	0.01 ng mL^−1^	[107]
ZrO_2_/rGO	ITO	voltammetry	CYFRA-21-1	2–22 ng mL^−1^	0.122 ng mL^−1^	[108]
TB/GO-COOH	GCE	voltammetry	CEA	0.5–60 ng mL^−1^	0.1 ng mL^−1^	[111]
PB/GO-COOH	GCE	voltammetry	AFP	0.5–60 ng mL^−1^	0.05 ng mL^−1^	[111]
PB/AuNPs/GO	GCE	voltammetry	AFP	0.01–100 ng mL^−1^	4.6 pg mL^−1^	[112]
CuS/GO	SPCE	voltammetry	AFP	0.001–10 ng mL^−1^	0.5 pg mL^−1^	[113]

rGO: reduced graphene oxide, PB: Prussian blue, CoHCF: cobalt hexacyanoferrate, TB: toluidine blue, GO-COOH: carboxylated graphene oxide, SPGE: screen-printed graphene electrode, SPCE: screen-printed carb electrode, SiC: silicon carbide, CYFRA-21-1: cytokeratin 19 fragment.
